# Supplementary material for: Modifiable and Non-Modifiable Risk Factors for Atherothrombotic Ischemic Stroke among Subjects in the Malmö Diet and Cancer Study
Source: Nutrients. 2021 Jun 6;13(6):1952. doi: 10.3390/nu13061952 (PMC8229981; doi:10.3390/nu13061952)
Supplement: Supplementary file 1 [file nutrients-13-01952-s001.zip › nutrients-1226787-supplementary.pdf]

Supplement Table S1. Hazard ratios (HR) and 95% confidence intervals (CI)[1] for incident atherothrombotic ischemic stroke by modifiable lifestyle risk factors among subjects with and without at-risk semi-modifiable comorbidities at baseline of the Malmö Diet and Cancer Study.

	**Hypertension**		**Dyslipidemia**		**Diabetes mellitus**		**Atherosclerotic disease**	
**Lifestyle factor**	Yes (N=16,211)	No (N=10,336)	Yes (N=6,780)	No (N=19,767)	Yes (N=1,111)	No (N=25,436)	Yes (N=602)	No (N=25,945)
Smoking								
Current	1.00 (ref)	1.00 (ref)	1.00 (ref)	1.00 (ref)	1.00 (ref)	1.00 (ref)	1.00 (ref)	1.00 (ref)
Former	0.63 (0.55-0.71)	0.63 (0.51-0.78)	0.64 (0.54-0.78)	0.61 (0.54-0.70)	0.66 (0.46-0.94)	0.62 (0.56-0.70)	0.73 (0.44-1.21)	0.62 (0.56-0.69)
Never	0.62 (0.55-0.70)	0.62 (0.50-0.75)	0.65 (0.54-0.78)	0.60 (0.53-0.68)	0.55 (0.38-0.80)	0.62 (0.56-0.70)	0.82 (0.44-1.53)	0.61 (0.55-0.68)
$P_{interaction}$#	0.839		0.542		0.197		0.410	
BMI								
Obese	1.00 (ref)	1.00 (ref)	1.00 (ref)	1.00 (ref)	1.00 (ref)	1.00 (ref)	1.00 (ref)	1.00 (ref)
Overweight	1.02 (0.89-1.16)	1.17 (0.83-1.63)	1.05 (0.87-1.26)	1.03 (0.87-1.21)	1.46 (1.04-2.06)	0.98 (0.86-1.12)	0.85 (0.51-1.44)	1.05 (0.92-1.19)
Normal-weight	0.95 (0.83-1.10)	1.17 (0.84-1.63)	0.93 (0.76-1.15)	1.01 (0.85-1.19)	0.97 (0.64-1.49)	0.96 (0.84-1.10)	0.80 (0.44-1.45)	1.00 (0.87-1.14)
$P_{interaction}$#	0.519		0.618		0.965		0.351	
Diet								
Low	1.00 (ref)	1.00 (ref)	1.00 (ref)	1.00 (ref)	1.00 (ref)	1.00 (ref)	1.00 (ref)	1.00 (ref)
Medium	0.89 (0.78-1.01)	0.91 (0.73-1.14)	0.93 (0.76-1.13)	0.88 (0.77-1.01)	0.85 (0.52-1.41)	0.90 (0.80-1.01)	0.83 (0.37-1.84)	0.90 (0.80-1.01)
High	0.85 (0.71-1.01)	0.78 (0.57-1.08)	0.89 (0.68-1.15)	0.81 (0.67-0.98)	0.80 (0.46-1.39)	0.84 (0.71-0.99)	1.14 (0.48-2.75)	0.82 (0.70-0.96)
$P_{interaction}$#	0.898		0.578		0.833		0.104	
Alcohol consumption								
Zero	1.22 (1.02-1.46)	0.94 (0.64-1.37)	1.20 (0.93-1.54)	1.14 (0.92-1.41)	0.79 (0.48-1.31)	1.22 (1.03-1.45)	1.77 (0.52-6.08)	1.15 (0.98-1.36)
Low	1.00 (ref)	1.00 (ref)	1.00 (ref)	1.00 (ref)	1.00 (ref)	1.00 (ref)	1.00 (ref)	1.00 (ref)
Moderate	0.90 (0.80-1.02)	0.91 (0.74-1.11)	0.88 (0.74-1.05)	0.92 (0.81-1.04)	0.86 (0.60-1.22)	0.91 (0.82-1.02)	1.00 (0.61-1.66)	0.90 (0.81-1.00)
High	1.04 (0.92-1.17)	0.87 (0.70-1.08)	0.98 (0.82-1.19)	1.00 (0.88-1.14)	0.93 (0.64-1.36)	1.00 (0.90-1.12)	1.19 (0.72-1.94)	0.98 (0.88-1.10)
$P_{interaction}$#	0.238		0.853		0.797		0.281	
Physical activity								
Low	1.00 (ref)	1.00 (ref)	1.00 (ref)	1.00 (ref)	1.00 (ref)	1.00 (ref)	1.00 (ref)	1.00 (ref)
Moderate	0.88 (0.78-0.99)	1.00 (0.82-1.23)	0.94 (0.80-1.11)	0.89 (0.78-1.01)	0.94 (0.67-1.31)	0.90 (0.81-1.00)	0.88 (0.53-1.47)	0.91 (0.82-1.01)
High	0.89 (0.79-0.99)	0.91 (0.74-1.12)	0.87 (0.73-1.03)	0.89 (0.79-1.01)	0.79 (0.55-1.11)	0.90 (0.81-1.00)	1.04 (0.63-1.74)	0.88 (0.80-0.98)
$P_{interaction}$#	0.760		0.747		0.435		0.624	

[1] Cox proportional hazards regression models with adjustment for non-modifiable risk factors (age, sex, stroke heredity score, and educational level) and mutual adjustment for the modifiable lifestyle risk factors and semi-modifiable co-morbidities presented in this table.

P-interaction is the p-value for the cross-product of the respective lifestyle risk factor and comorbidity included in the model.
